# Germination responses of *Lens Culiunaris* L. seeds to osmotic potentials at cardinal temperatures using hydrothermal time model

**DOI:** 10.1186/s12870-024-05223-0

**Published:** 2024-06-05

**Authors:** Ibrar Ullah, Sami Ullah, Fazal Amin, Jehad S. Al-Hawadi, Mohammad K. Okla, Ibrahim A. Alaraidh, Hamada AbdElgawad, Ke Liu, Matthew Tom Harrison, Shah Saud, Shah Hassan, Taufiq Nawaz, Mo Zhu, Haitao Liu, Shah Fahad

**Affiliations:** 1 Department of Botany, University of Peshawar, Peshawar, 25120 Pakistan; 2 Faculty of Science, Zarqa University, Zarqa, 13110 Jordan; 3 Botany and Microbiology Department, College of Science, King Saud University, P.O. Box 2455, Riyadh, 11451 Saudi Arabia; 4 Integrated Molecular Plant Physiology Research, Department of Biology, University of Antwerp, Antwerp, 2020 Belgium; 5 Tasmanian Institute of Agriculture, University of Tasmania, Burnie, TAS 7250 Australia; 6 College of Life Science, Linyi University, Linyi, 276000 Shandong China; 7 Department of Agricultural Extension Education & Communication, The University of Agriculture, Peshawar, 25130 Khyber Pakhtunkhwa Pakistan; 8 Department of Biology and Microbiology, South Dakota State University, Brookings, SD 57007 USA; 9 College of Life Sciences, Henan Normal University, Xinxiang, 453007 P.R. China; 10 Henan International Joint Laboratory of Agricultural Microbial Ecology and Technology, Henan Normal University, Xinxiang, 453007 P.R. China; 11 Xinxiang Key Laboratory of Plant Stress Biology, Xinxiang, 453000 P.R. China; 12 College of Resources and Environment, Henan Agricultural University, Zhengzhou, 450002 PR China; 13 Department of Agronomy, Abdul Wali Khan University Mardan, 23200 Khyber Pakhtunkhwa, Pakistan

**Keywords:** Lentil, Hydrothermal time model, Germination, Osmotic potential, Temperature

## Abstract

**Background:**

Lentil is a significant legume that are consumed as a staple food and have a significant economic impact around the world. The purpose of the present research on lentil was to assess the hydrothermal time model’s capacity to explain the dynamics of *Lens culinaris* L. var. Markaz-09 seed germination, as well as to ascertain the germination responses at various sub-optimal temperatures (T) and water potentials (Ψ). In order to study lentil seed germination (SG) behavior at variable water potentials (Ψs) and temperatures (Ts). A lab experiment employing the hydrothermal time model was created. Seeds were germinated at six distinct temperatures: 15 ^0^С, 20 ^0^С, 25 ^0^С, 30 ^0^С, 35 ^0^С, and 40 ^0^С, with five Ψs of 0, -0.3, -0.6, -0.9, and − 1.2 MPa in a PEG-6000 (Polyethylene glycol 6000) solution.

**Results:**

The results indicated that the agronomic parameters like Germination index (GI), Germination energy (GE), Timson germination index (TGI), were maximum in 25 ^0^C at (-0.9 MPa) and lowest at 40 ^0^C in 0 MPa. On other hand, mean germination time (MGT) value was highest at 15 ^0^C in -1.2 MPa and minimum at 40 ^0^C in (-0.6 MPa) while Mean germination rate (MGR) was maximum at 40 ^0^C in (0 MPa) and minimum at 15 ^0^C in (-0.6 MPa).

**Conclusions:**

The HTT model eventually defined the germination response of *Lens culinaris* L. var. Markaz-09 (Lentil) for all Ts and Ψs, allowing it to be employed as a predictive tool in *Lens culinaris* L. var. Markaz-09 (Lentil) seed germination simulation models.

## Background

Lentil is a pulse that is eaten around the world [1]. It is a pulse crop that has been used in agriculture for much of human history. Canada produces 33% of the world’s lentils, whereas India produces 25%. Other important countries include the United States, Turkey, Nepal, Australia, Nepal, and Pakistan, among others [2]. Humans perceive lentils to have a high nutritious content and health advantages [3]. Lentils contain carbohydrates, minerals, vitamin B, iron, magnesium, copper, selenium, potassium, zinc, thiamin, and folate. riboflavin, pantothenic acid, niacin and fiber, in addition to a high protein content [4]. Lentils can be used to treat a lot of health problems, including Coronavirus disease 2019, managing blood sugar abnormalities, lowering blood lipids, and lowering the risk of cardiovascular disease and cancer [5, 6].

Emergence and germination are the utmost crucial phases in a plant’s life cycle because they influence how effectively plants employ the water and nutrients resources accessible to them [7]. Temperature, pH, soil moisture and light are all known to have an impact on germination of seed [8]. The seed germination’s frequency and the dispersal of species are significantly affected by environmental temperatures [9]. Throughout the germination phase, seed is the unit of reproduction. As a result, a basic knowledge of seed germination is crucial for the production of crops, particularly in a world that is intensely aware of the fragile balance between the global population and food production [10].

Hydrothermal time model (HTT) is a mathematical model that can be used to quantify and describe the combined impacts of water potential (Ψ) and temperature (T) on biological activities (Allen 2003). The degree to which ambient temperature and water potential conditions surpass specified base or threshold values is inversely related to the time required to complete for a certain fraction of a population. When T goes below the base temperature (Tb) or Ψ falls below (i.e., is more negative than) the base water potential (Ψb), the process is hindered. Ψb and Tb heterogeneity account for differences in completion time among members of a community. HTT is a population-based threshold-type model, to put it another way. It was created to describe seed dormancy and germination and has virtually solely been utilized for that purpose thus far [11].

The present study’s goal was, (1) to determine the efficiency of the hydrothermal time model in studying seed germination of *Lens culinaris* L. var Markaz-09 at various Ts and Ψs, (2) to establish the SG at cardinal Ts and various water potentials.

## Material and method

### Experiment protocol and seed description

Lentil (*Lens culinaris* L.) var. Markaz-09 seed with a 95.0% viability rate were brought from the NARC (National Agriculture Research Centre, Islamabad, Pakistan). The seeds surface sterilization was done for 3 min with 0.5 g of mercuric chloride and 95 ml of distilled water solution, dried at room temperature after being washed with distilled water. An experiment using randomized complete block design (RCBD) was carried out in Petri dishes in the Laboratory of Plant Physiology in the Botany department at the University of Peshawar in Khyber Pakhtunkhwa, Pakistan. The studies were conducted at six different temperatures (15 ^0^С, 20 ^0^С, 25 ^0^С, 30 ^0^С, 35 ^0^С, and 40 ^0^С) with five Ψs of 0, -0.3, -0.6, -0.9, and − 1.2 MPa. To prepare the solution with various Ψs, PEG6000 (polyethylene glycol; Merck, Germany) was utilized [12]. In each Petri dish, 10 seeds were put on Withman No. 1 filter paper and steeped in 10 ml distil water and PEG solutions. Except for the measurement periods, the Petri dishes were kept in an incubator (Memmert Beschickung-Loading-Model 100–800, Schwabach, Germany). Every treatment is triplicate [13]. The measurement was taken after the radicle length reach 1 mm.

### Data analysis

The HTT, HT, and TT models were analyzed and measured using a repeated probit regression analysis [10, 14]. The GR for the 50th percentile of germination was calculated using the inverse of germination time for each percentile at each Ψ or T.

#### Thermal time (TT)

There are mathematical models that describe how temperature affects germination patterns [11]. According to this model, the germination rate (GRg, or 1/tg) for a specific seed fraction, percentage, or germination period should be a linear function of temperature above base temperature. The minimum temperature at which germination may take place is known as the base temperature or minimum (Tb). Temperature on which germination proceeds most quickly is referred to as the optimum temperature. This can be written as: for sub-optimal temperatures:

$$\theta T1=TTsub=(T-Tb) t$$

For supra-optimal temperatures:

$$\theta T2=TTsupra=(T-Tb) t$$

Due to the fact that germination rate (GR) is inversely proportional to radicle emergence time, which may be written as:

$$GR\left(g\right) = 1/T\left(g\right)= 1/\theta H(T-Tb)$$

The thermal time constants θT1 and θT2 and T is the real temperature and Tb express the base temperature. GR stands for the population’s average germination rate (g).

#### NumberedHydro time (HT)

The hydro time notion was proposed first time by [15]. The base or threshold value (Ψb) will only prevent a percentage of the seed population (g) from germinating. As accordance to the hydro time model, the rate of germination is directly proportional to Ψ. The hydro time constant (θH) is represented by the following formula:

$$\theta H\left(g\right) = (\varPsi ? \varPsi b) tg$$

$$GR = 1/tg = (\varPsi -\varPsi b)/\theta H$$

tg is the period for radicle emergence, GR (g) represents the germination rate, θH represents hydro time constant, Ψ is the real osmotic potential, and Ψb is the germination fraction’s base water potential.

#### Hydrothermal time model (HTT)

The HTT which may depict pattern of seed germination, was created by combining the aforementioned hydro-time models and thermal-time. Combining hydro time equations with thermal time equations allows for the definition of a hydrothermal time constant (θHT) at sub-optimal temperatures (T) [11, 16]. The Hydrothermal time model is expressed at Ts ≤ To [15]:

$$\theta HT = (\varPsi - \varPsi b) (T-Tb)tg$$

$$Probit \left(g\right)= [\varPsi ? (\theta H/tg) - \varPsi b(50)/\sigma \varPsi b$$

Where Ψb (50) is the midpoint of Ψb. θHTT illustrate the hydrothermal time constant (MPa h). While σΨb is the standard deviation in Ψb.

### Germination and agronomic parameters

The germination parameters presented below were estimated using the germination on each day, root and shoot lengths, dried and fresh weights of the germinated seeds.

#### Germination percentage (G%)

The formula of [17] used to calculate this germination percentage.

$$Germination percentage \left(G\text{\%}\right) = Ne/Nt\times 100$$

Where Nt shows the total number of seeds sown and Ne shows the number of seedlings that emerged.

#### Germination energy (GE)

The formula of [18] used to compute the germination energy.

$$GE = X1/Y1 + (X2-X1)/Y2+(Xn-Xn-1)/Yn$$

The symbol X1. X2 and Xn in the equation above shows the count of seed that are germinated on the 1st day, 2nd day, and so 4th. Whereas Y1, Y2 and Yn stand for the first, second, and last day of germination.

#### Mean germination time (MGT)

MGT was calculated using the formula of [19, 20].

$$MGT= \text{?}fx/\text{?}f$$

#### Germination index (GI)

The Germination index gives info on the rate and percentage of germination. The germination index was calculated using the procedure provided by [21]

$$\text{G}\text{I} = (10\times \text{n}1) + (9\text{n}\times \text{n}2) + \dots \dots \dots \dots + (1\text{n}\times \text{n}10)$$

The number of seed that are germinated on day 1, 2, and 10 was shown by the symbols n1, n2,…, and n10. Whereas 10, 9, and 1 show the weighted average of seed number that germinated on day.

#### Germination rate index (GRI)

Greater and maximum GR are indicated by higher GRI values, which also represent the percentage of regular SG during the germination phase [22].

$$GRI =G1/1+G2/2+G3/3\dots . +Gx/x$$

Where G1 shows the proportion of seed that germinated on the 1st day after sowing, G2 shows the proportion of seed that germinated on the 2nd day following sowing, and so on.

#### Timson germination index (TGI)

The TGI represents the daily average of germinated seeds. TGI was determined using the methodology of [23].

Timson Germination Index (TGI) = ϵG ÷ T.

#### Seed vigor index-I (SVI-I)

From each pot length of three seedling were measured and calculation was done using the [24] formula.

SVI-I = Seedling length (cm) × seed germination % age.

#### Seed vigor index-ii (SVI-II)

Using an electrical balance, the dry weight of three seedling from each pot was measured, and the percentage of seed germination was multiplied in the manner suggested by [25].

SVI-II = seedling dry weight (mg) × seed germination %age.

#### Mean moisture content (MMC)

The mean moisture content was calculated using the formula stated below [13].

M.M.C = (Fresh weight – Dry weight) ÷ dry weight.

#### Mean germination rate (MGR)

The below given formula was used to discover MGR [10].

Mean germination rate = 1/Mean germination time.

### Antioxidant enzymes activities

#### Ascorbate peroxidase (APX) activity

After centrifuging 0.5 g of fresh plant material with 10 milliliters of phosphate buffer, the resulting supernatant was collected. The final volume was brought down to 3.0 ml by adding deionized water after the supernatant, which was 0.1 ml in volume, was combined with 0.5 mM ascorbic acid and 0.1 mM EDTA. Following the addition of 0.1 milliliters of hydrogen peroxide to the mixture, the absorbance was measured at 290.0 nanometers using the protocol of W Shah, S Ullah, S Ali, M Idrees, MN Khan, K Ali, A Khan, M Ali and F Younas [26].

#### Superoxide dismutase (SOD) activity

The mixture was centrifuged after 0.5 g of fresh plant material were chopped with 5 milliliters of phosphate buffer. The supernatant was collected after the mixture was centrifuged. 1 milliliter of riboflavin was added to 0.1 milliliter of supernatant, which was then mixed with EDTA at a concentration of 3 mill molar, 25 µl of nitro tetrazolium blue chloride, 5 milliliters of methionine, and Na2CO3. After that, the mixture was stored at room temperature for protection. It was observed that the absorbance was at 560.0 nm and Superoxide dismutase activity was measured according to G Lalay, S Ullah and I Ahmed [27].

#### Peroxidase activity (POD)

The method of S Uddin, S Ullah and M Nafees [28] was tracked for the peroxidase activity investigation in fresh plant material. The supernatant was collected after 0.5 g of plant-fresh material was chopped and placed in 2 milliliters of 2-(N-Morpholino) ethanesulfonic acid (MES). The mixture was then centrifuged. In order to treat 0.1 ml of supernatant, 1.5 ml of 100 mM MES, 0.1 ml of phenylenediamine, and 0.04 ml of hydrogen peroxide are added. A measurement of absorbance was taken at 485.0 nm.

#### Catalase (CAT) activity

The Catalase activity was examined using the method described by of S Ullah and A Bano [29]. Fresh plant tissues weighing 0.5 g were combined with 10 milliliters of phosphate buffer, filtered, centrifuged, and the supernatant was then collected. 0.1 ml of supernatant was mixed with 0.5 ml of H_2_O_2_ and the absorbance was measured at 240.0 nm.

#### Guaiacol peroxidase (GPX) activity

Fresh plant tissues weighing 0.5 g were combined with 10millilitres of phosphate buffer, centrifuged, and the supernatants was then extracted. 0.1 ml of supernatant was mixed with guaiacol (16 mM) and phosphate buffer (50 mM), then 2 mM H_2_O_2_ was added. The mass of the reaction mixture was modified to 3 ml by the addition of deionized water. The absorbance was recorded at 470 nm and according to the protocol of M Nafees, S Ullah and I Ahmed [30].

### Statistical analysis

We studied the impacts of thermal time, hydro time, and their interaction (hydro-thermal time model) on germination characteristics and seed germination rate using SPSS Statistic 25 (IBM) and Sigma Plot Version 10.0. Excel software was employed to do the fundamental statistical computations. The linear probit regression analysis was used in SPSS statistic 25 to compute the value of the following given parameters: σΨb, Ψb (50), R2, SE, F, T-test, and Sig. Graphs of germination fraction and germination parameter against Ψ and T were made using Origin 2021 PC Corporation. The data analysis techniques of correlation analysis, histogram generation, and principal component analysis (PCA) were carried out using the Origin Pro software.

## Results

### Effect of osmotic potential and cardinal temperature on agronomic attributes

**Fig. 1:**
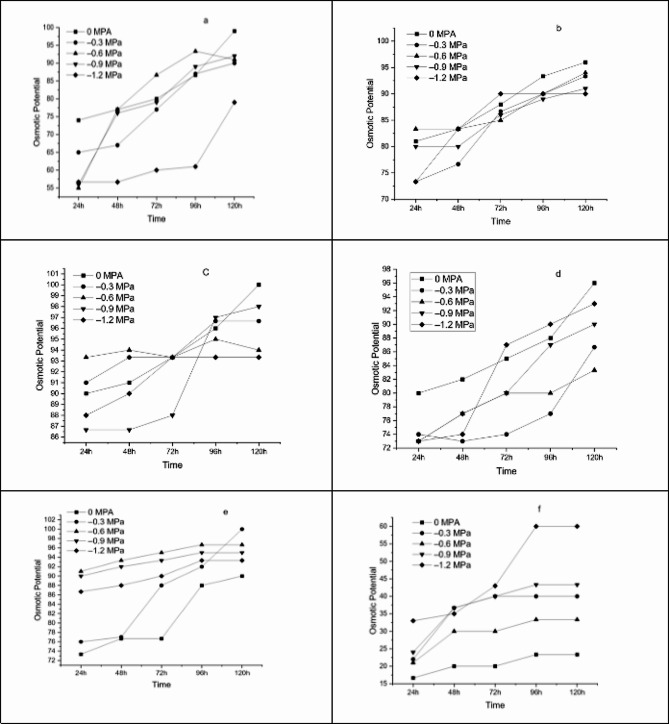
Germination for *Lens culiunaris* var. Markaz-09 at (**a**) 15 °C, (**b**) 20 °C, (**c**) 25 °C, (**d**) 30 °C, (**e**) 35 °C and (**f**) 40 °C having different osmotic potentials (0 MPa, -0.3 MPa, -0.6 MPa, -0.9 MPa and − 1.2 MPa)

**Fig. 2:**
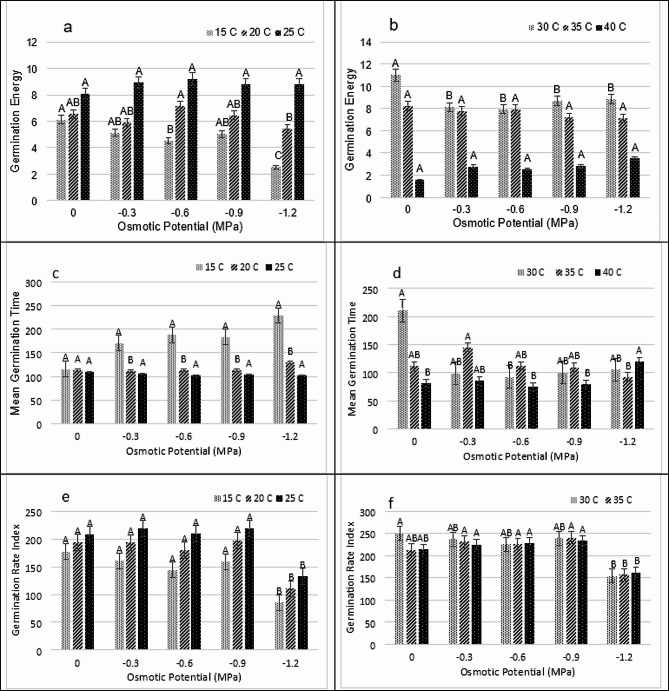
Impact of water potentials and temperatures on **(a and b)** Germination Energy, **(c and d)** Mean Germination Time and **(e and f)** Mean Germination Rate of *Lens culiunaris L.* var. Markaz-09 using hydrothermal time model

The germination rate and seed percentage were initially favoured by an increase in temperature amplitude, but this fall after T hit a particular threshold. GP was highest at 35 ° C and minimum at 15 and 40 ° C (Fig. 1A, B, C, D, E and F). The lowest values of GP, 10% and 13.33% were recorded at 15 and 40˚C under (-1.2 MPa and 0 MPa respectively), while maximum 100% at 35˚C under (0 MPa) *Lens culinaris* L. var. Markaz 09 respectively. Germination energy (GE) were maximum at 25 ^0^C in (-0.9 MPa) and minimum at 40 ^0^C in (0 MPa). The value MGT was highest in 15 ^0^C at (-1.2 MPa) and lowest at 40 ^0^C in (-0.6 MPa), while the GRI was maximum at 40 ^0^C. (Figure 2A, B, C, D, E and F)

**Fig. 3:**
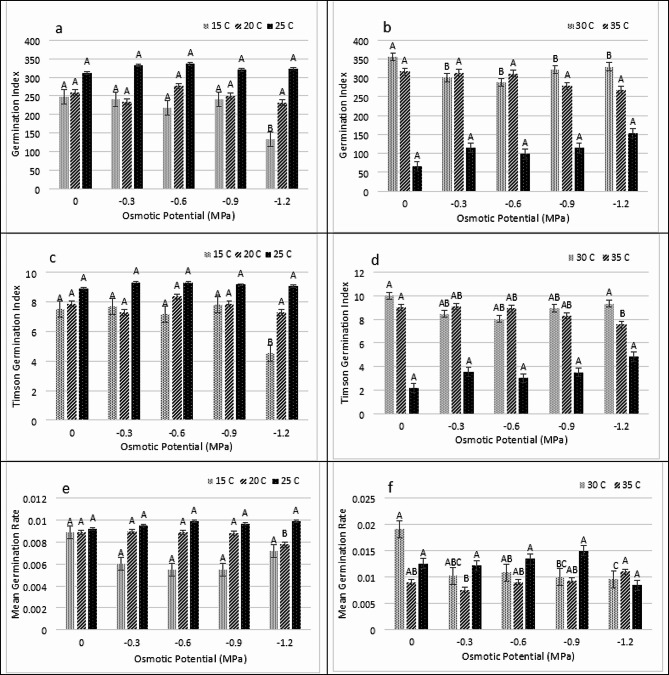
Impact of water potentials and temperatures on **(A and B)** Germination Index, **(B and C)** Timson Germination Index and **(E and F)** Germination Rate Index of *Lens culiunaris L.* var. Markaz-09 using hydrothermal time model

**Fig. 4:**
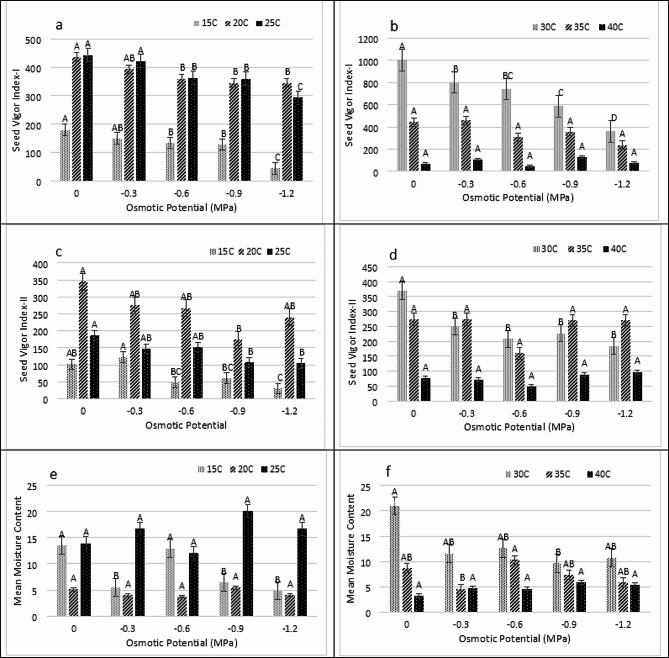
Impact of water potentials and temperatures on **(a and b)** Seed Vigor Index-I, **(c and d)** Seed Vigor Index-II and **(E and F)** Mean Moisture Content of *Lens culiunaris L.* var. Markaz-09 using hydrothermal time model

The GI (Germination index) and TGI (Timson germination index) were maximum at 25 ^0^C in (-0.9 MPa) and lowest in 40 ^0^C at (0 MPa). On other hand Mean germination rate (MGR) was highest at 40 ^0^C in (0 MPa) and lowest at 15 ^0^C in (-0.6 MPa) (Fig. 3A, B, C, D, E and F). The SVI-II and SVI-I were maximum at 30 ^0^C in (0 MPa) and lowest at 40 ^0^C in (-0.6 MPa). On the other hand, the MMC value was maximum in -0.6 MPa at 30 ^0^C (Fig. 4A, B, C, D, E and F).

### Effect of osmotic potential and cardinal temperature on antioxidant enzymes

**Fig. 5:**
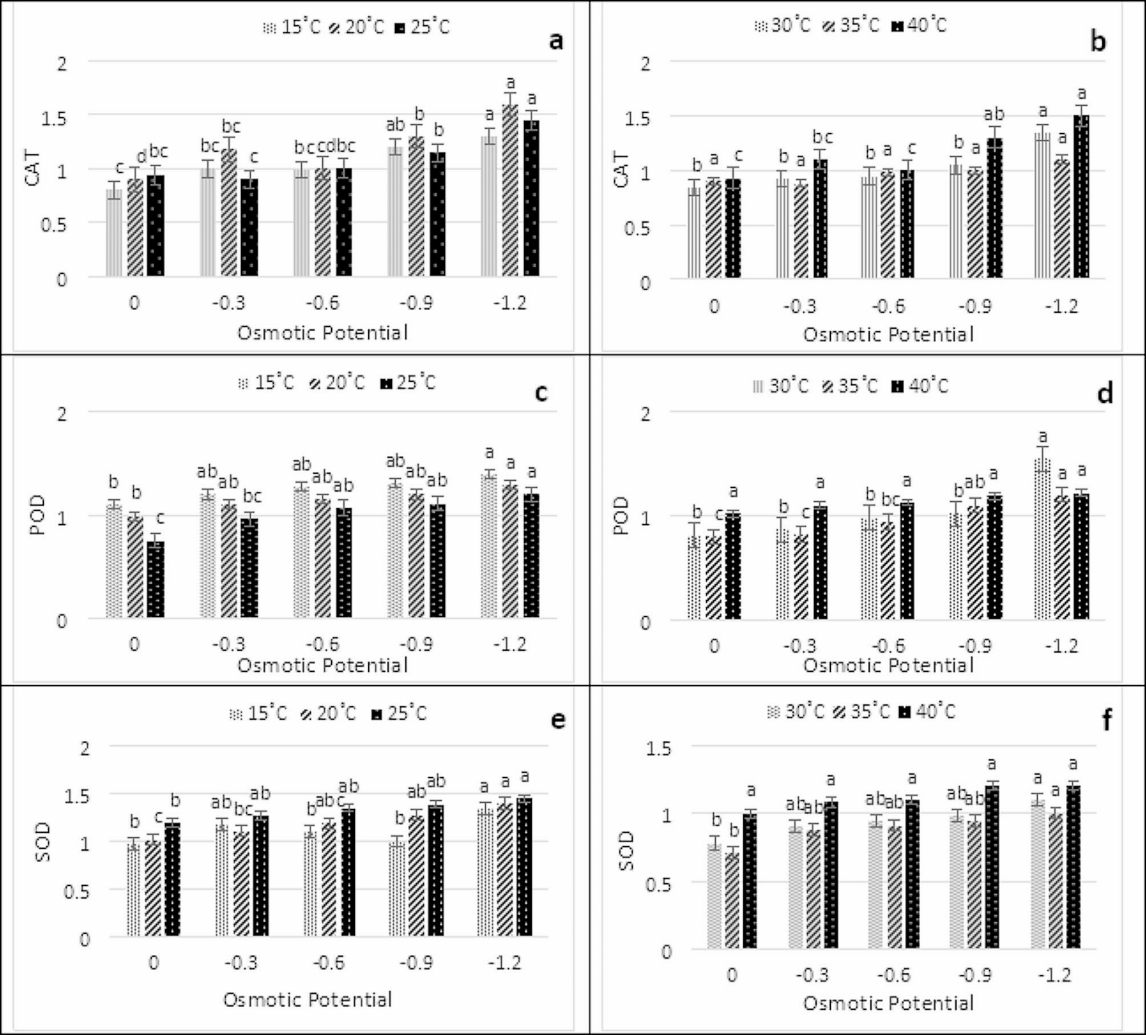
Interactive effect of water potential, temperature on antioxidant enzymes (**a and b)**-CAT, **(c and d)**-POD, **e and f)**-SOD under PEG induced stress at different temperatures (15 °C, 20 °C, 25 °C, 30 °C, 35 °C and 40 °C)

**Fig. 6:**
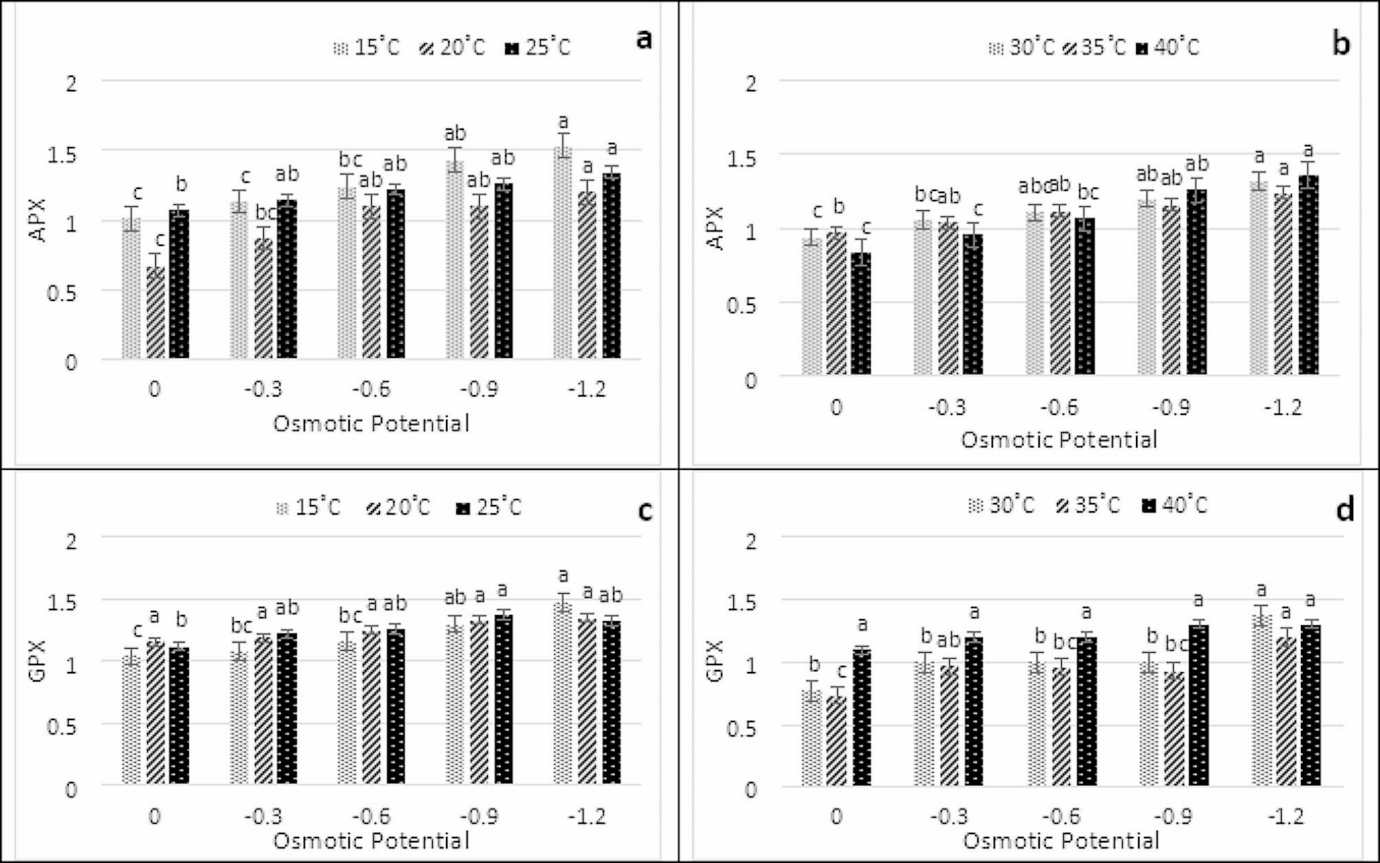
Interactive effect of water potential, temperature on antioxidant enzymes (**a** and **b**-APX) and (**c** and **d**-GPX) under PEG induced stress at different temperatures (15 °C, 20 °C, 25 °C, 30 °C, 35 °C and 40 °C)

The findings regarding antioxidant enzyme indicated that the quantity of antioxidant enzyme in fresh plant tissues was substantially impacted by temperature and osmotic potential fluctuations. The findings presented in Fig. 5a-f indicate that the CAT exhibited its maximum activity at 15ºC at -1.2 MPa, whereas it’s minimum value was documented at 0 MPa at 15 °C. In a similar vein, the POD activity at 30 °C peaked at -1.2 MPa, while the lowest activity was observed in the control group at 25 ºC (Fig. 5a-f). Similarly, at 25 ºC, the SOD activity peaked at -1.2 MPa, with the lowermost value being − 0 MPa at 35 ºC (Fig. 5a-f). As shown in Fig. 6a-d APX and GPX have demonstrated their maximum values at -1.2 MPa and 15 °C, respectively, with APX reaching its minimum value at 0 MPa at 20 °C and GPX reaching its minimum value at 35 °C at 0 MPa. It has been observed that all enzymes exhibited normal activity within the temperatures range of 25–30 °C and 0 MPa. Nevertheless, both the greatest and lowest treated temperatures exhibited an adverse effect. When examining the thermal and osmotic responses, it was observed that APX and GPX exhibited the most notable response at 20 °C and − 1.2 MPa, respectively, as illustrated in Fig. 6a-d. Moreover, at 0Mpa, the minimum response was observed for all antioxidant enzymes.

**Fig. 7:**
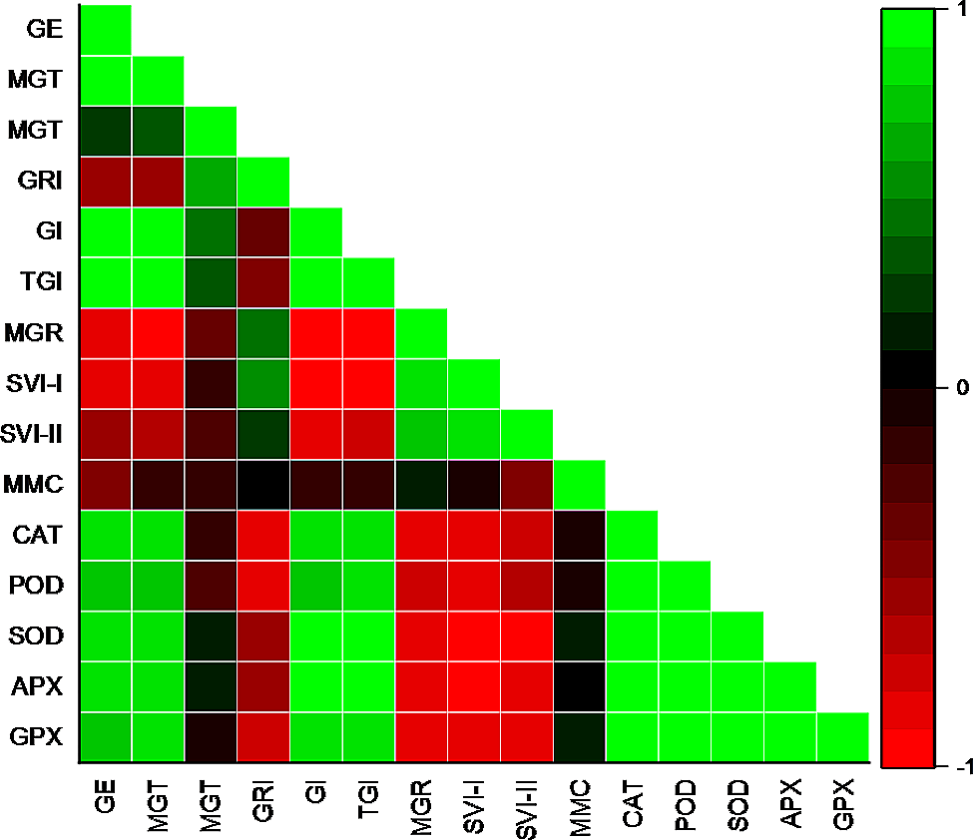
Correlation between various germination attributes of *Lens culiunaris* L. var. Markaz-09 using hydrothermal time model

**Fig. 8:**
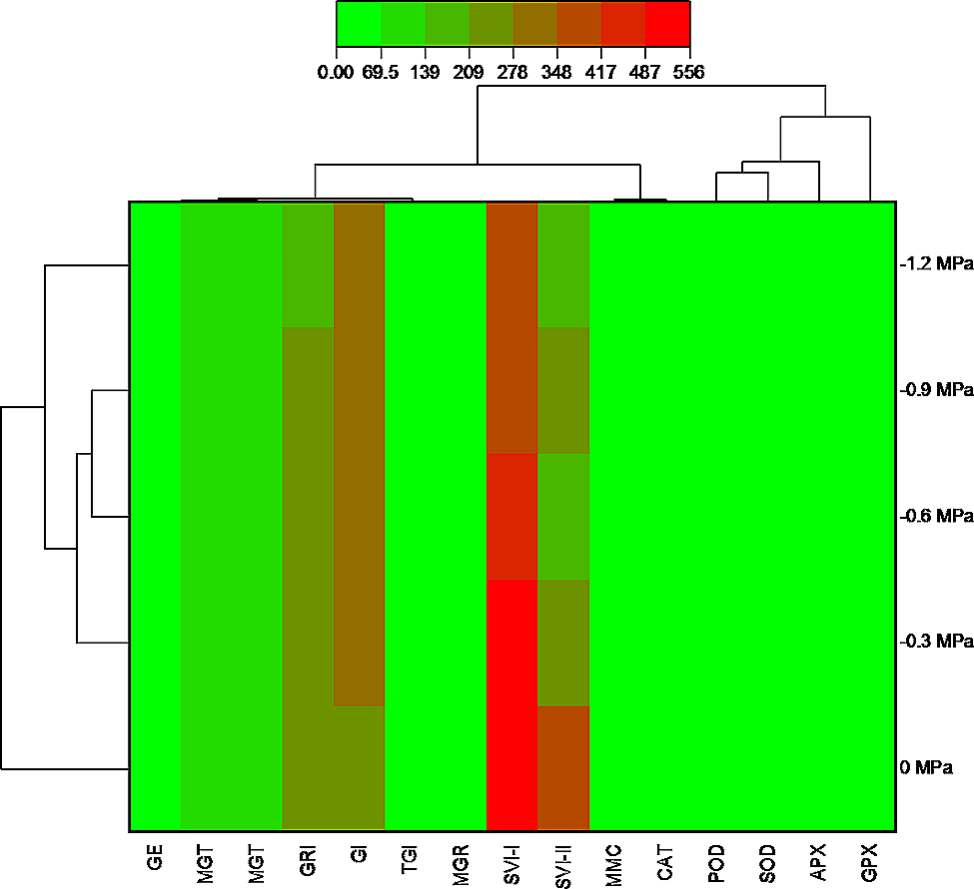
Heatmap histogram correlation between various germination attributes of *Lens culiunaris* L. var. Markaz-09 using hydrothermal time model

**Fig. 9:**
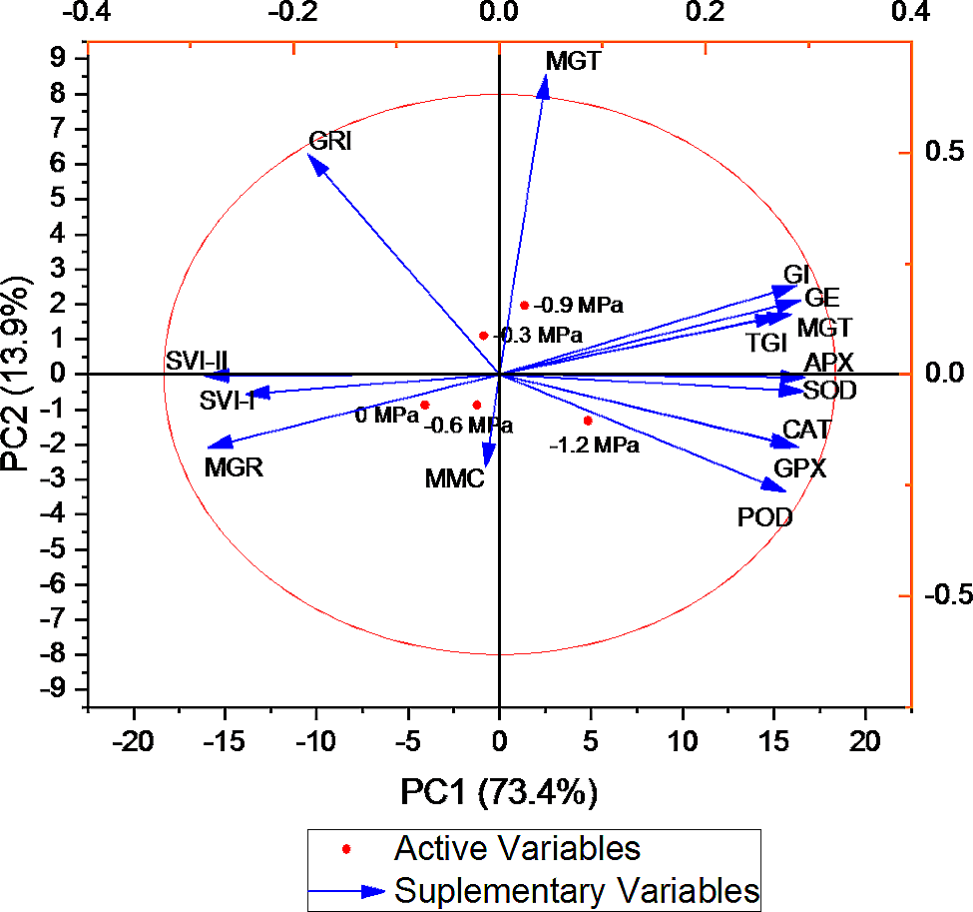
Loading Plot of Principal component analysis (PCA) on various germination attributes of *Lens culiunaris* L. var. Markaz-09 using hydrothermal time model

A negative correlation was observed between GE and GRI, MGR, SVI-II, SVI-I, and MMC, whereas GE was positively correlated with MGT, GI, and TGI (Fig. 7). The GI exhibits a negative correlation with SVI-I, SGR, and SVI-II, while its correlation with TGI is positive. There exists a positive correlation among all enzymes. As illustrated in Fig. 8, two distinct clusters are observed to form between treatments. The initial cluster comprises the treatment at 0 MPa, whereas the subsequent cluster comprises the control, -0.3 MPa, -0.6 MPa, -0.9 MPa, and − 1.2 MPa. The germination dataset was analyzed using PCA. The findings indicate that every treatment is substantially dispersed across the dataset. The analysis of the treatment distribution indicates that the germination properties were significantly influenced by the osmotic potential. 73% of the total variance was accounted for by the first two components, according to the PCA results. As the variation in the first two components was the greatest, a biplot based on PCA was generated (Fig. 9).

The results of HTT experiment forecast that the water potential and temperature had significantly control the germination parameters such as PL (plumule length), G%, radicle length, GI, TGI, MMC, SVI-I, GE, SVI-II, MGT and MGR, in comparison with control treatment of Lentil (*Lens culinaris* L. var Markaz 09). According to Table 1 the temperature and water potential had significantly (*P* ≤ 0.05) affected GR (germination rate) and germination percentage (G%) of *Lens culinaris* L. var. Markaz 09. It showed that germination was increased from 10 to 100% with rising of temperature from 15 ˚C to 35˚C, then the value decreased second time to 13.33% as the temperature surpass 35˚C (optimum T) to 40˚C of *Lens culinaris* L. var. Markaz 09. The result also shows that the highest θT1 value was reported in 35˚C at -0.3 MPa and minimum at 15 ˚C in -1.2 MPa (Table 1). On the other side the highest value of θT2 was reported at -0.3 MPa in 15˚C and minimum in 40 ˚C at (0 MPa).

The TT theory is thought to be well suitable to germination data in distil water, with R^2^ growing by 0.41. The hydrothermal time model may be applied to investigate the influence of temperature and water potential above the thermal and hydro thresholds on seed germination. The HTT concept has a higher value (R2 = 0.41 at 30 C) at sub-optimal temperature (T < T0) than at supraoptimal T (R2 = 0.24). T and Ψ interaction have significant effect on G% and GR (*P* < 0.05). According to the HTT model’s comparing results, the maximum HTT value was discovered in 35 °C at 0 MPa (Table 2).

The base temperature or minimum temperature (Tb) in our experiment was taken 15 ◦C, below from this temperature the growth of the seed is very slow and all plant will find it challenging to maintain its physiological functions. 25 to 30 ^o^C was the ideal temperature range for the plant to grow at its fastest rate. The growth of the plant was reduced above the optimum temperature and the lowest growth was detected at 40 ^0^C in our experiment (Table 3).

**Table 1:** The estimated parameters of the hydro and thermal time models to describe *Lens culiunaris L.* var. Markaz-09 seed germination under fluctuating different temperatures (Ts) and water potentials (ψs)

Temperature	Treatment	TTsub/θT1	TTsupra/θT2	θH(MPa/h)	θHTT (MPa/h)	TT GR	HT GR
15^o^C	0 MPa	306.40	1838.40	91.92	459.60	0.016	0.016
	-0.3 MPa	336.00	2016.00	101.10	403.20	0.015	0.012
	-0.6 MPa	324.00	1944.00	96.60	291.60	0.015	0.009
	-0.9 MPa	346.40	2078.40	103.02	207.84	0.014	0.006
	-1.2 MPa	204.00	1224.00	60.80	61.20	0.058	0.011
20 ^o^C	0 MPa	656.00	1640.00	98.40	984.00	0.015	0.015
	-0.3 MPa	630.40	1576.00	94.56	756.48	0.016	0.013
	-0.6 MPa	696.00	1740.00	104.40	626.40	0.014	0.009
	-0.9 MPa	691.20	1728.00	103.68	414.72	0.014	0.006
	-1.2 MPa	633.60	1584.00	95.04	190.08	0.016	0.003
25 ^o^C	0 MPa	1000.80	1334.40	100.08	1501.20	0.015	0.015
	-0.3 MPa	1029.60	1372.80	102.96	1235.52	0.015	0.012
	-0.6 MPa	1008.00	1344.00	100.80	907.20	0.015	0.009
	-0.9 MPa	1051.20	1401.60	105.12	630.72	0.014	0.006
	-1.2 MPa	1000.80	1334.40	100.08	300.24	0.015	0.003
30 ^o^C	0 MPa	1116.80	837.60	83.76	1675.20	0.018	0.018
	-0.3 MPa	1244.80	933.60	93.36	1493.76	0.016	0.013
	-0.6 MPa	1168.00	876.00	87.60	1051.20	0.017	0.010
	-0.9 MPa	1296.00	972.00	97.20	777.60	0.015	0.006
	-1.2 MPa	1388.80	1041.60	104.16	416.64	0.014	0.003
35 ^o^C	0 MPa	1780.00	712.00	106.80	2670.00	0.014	0.014
	-0.3 MPa	1708.67	683.47	102.52	2050.40	0.015	0.012
	-0.6 MPa	1657.33	662.93	99.44	1491.60	0.015	0.009
	-0.9 MPa	1653.33	661.33	99.20	992.00	0.015	0.006
	-1.2 MPa	1364.67	545.87	81.88	409.40	0.019	0.004
40 ^o^C	0 MPa	470.40	78.40	23.52	705.60	0.103	0.103
	-0.3 MPa	844.80	140.80	42.24	1013.76	0.064	0.051
	-0.6 MPa	691.20	115.20	34.56	622.08	0.068	0.041
	-0.9 MPa	888.00	148.00	44.40	532.80	0.063	0.025
	-1.2 MPa	1248.00	208.00	62.40	374.40	0.025	0.005

**Table 2:** Estimation of hydrotime model parameters for *Lens culiunaris L.* var. Markaz-09 using non-linear regression

Temperature	ѱb(50) (MPa)	σψb (MPa)	*R* ^2^	SE	F	T	Sig.
15˚C	-1.25	0.32	0.14	4.15	2.12	7.91	0.17
20˚C	-1.39	0.09	0.00	1.28	0.04	23.20	0.85
25˚C	-1.39	0.11	0.00	1.40	0.02	21.81	0.88
30˚C	-1.27	0.13	0.41	1.48	9.08	0.41	0.01
35˚C	-1.33	0.16	0.41	1.78	8.97	19.35	0.01
40˚C	-0.59	0.34	0.24	3.98	4.04	2.01	0.07

**Table 3:** Estimated germination and cardinal temperature values for *Lens culiunaris L.* var. Markaz-09 using the hydrothermal time model

Hydrothermal time model parameters		
Variables		Lens culiunaris L
b (50) (MPa)		-1.20
σ*ψ*b (MPa)		0.19
θH (MPa˚Ch-1)		96.77
kT (MPa˚Ch-1)		0.104
**Cardinal temperatures**		
*T*b (˚C)		15
*T*o (˚C)		30
*T*c (˚C)		40
*R* ^*2*^		0.200

kT = Thermal Energy of the seed, T_o_ = optimum temperature of the seed, T_b_ = base temperature of the seed, T_c_ = ceiling temperature of the seed

## Discussion

It is possible to evaluate and quantify the effect of various abiotic variables on the time of SG in different seed lots using the TT, HT, and HTT models [31]. Among these abiotic factors, temperature as well as osmotic potential are the most influential environmental variables on seed germination in a wide variety of plants [31, 32]. Likewise, the outcomes of our investigation demonstrated that both temperature and osmotic potential significantly impacted the process of seed germination.

Temperature response of seed can be characterized in general by theirs cardinal Temperatures (i.e., Tb, To and Tc). Our experiment’s cardinal temperature was found to be (15 ^o^C, 30 ^o^C and 40 ^o^C for Tb, T0, and Tc) respectively (Table 3). The result showed that G% was maximum at 35 °C in 0 MPa, while the lowest germination was recorded at 40 °C in 0 MPa. The decrease in germination % may be caused by the high temperature denaturation of critical amino acids [33]. In comparison with control, the maximum GP was recorded at 30 in -1.2 MPa. This suggests that a variety of plant species’ GP and GR are influenced by temperature, which is a key element in seed germination.

An additional factor that exerts an impact on seed germination is water potential. Furthermore, our investigation revealed that water potential had a significant influence on the germination of seeds. At 35 0 C, the G% was highest in control and lowest in -1.2 MPa. At other temperature, the same effect was predicted, demonstrating that reducing in lowered in G%. Reduced in caused the water supply to the seed to be less sufficient for germination. This result is similar with the studies of [12, 33] and [34] for wheat, watermelon and zucchini.

We found that a reduction in osmotic potential (towards negativity) significantly increased the GR values (*p* ≤ 0.01) for all cardinal temperatures (Table 1). GR decreased when the osmotic potential was decreased relative to the control. The experiment recorded a minimum temperature (Tb) of 15 degrees Celsius, below which the germination rate exhibited a decline. 30 degrees Celsius was the ideal temperature (To) for germination, whereas 40 degrees Celsius was the maximum temperature (Tc) that induced physiological and biochemical activity in plants. This is comparable to [13] which states that there are three cardinal temperatures (Ts) that are essential in delineating the germination characteristics of seed and determine the temperature required for germination.

Due to water stress, the concentration of antioxidant enzymes such as superoxide dismutase (SOD), peroxidase (POD), and catalase (CAT) decreases. To mitigate cellular harm, the antioxidant system diminishes reactive oxygen species (ROS) accumulation through enzymatic scavenging of ROS and elevation of antioxidant concentrations such as APX and GPX [35]. SOD is a critical component of the antioxidant defense mechanism as it functions as the primary barrier against superoxide radicals. SOD-catalyzed dismutation of reactive oxygen species (ROS) generates H2O2 as a reaction product, which is subsequently scavenged by CAT and APOX [36]. The concentrations of guaiacol peroxides (GPX) and ascorbate peroxidase (APX) were diminished in the presence of water deficiency stress. APX, a crucial antioxidant, is accountable for the elimination of reactive oxygen species (ROS) in the presence of oxidative stress. Ascorbate peroxidase facilitates the conversion of H_2_O_2_ to regular water by employing ascorbate as a donor of electrons and catalyzing the reaction. It is imperative to acknowledge that the regulation of APX expression varies as a consequence to environmental stresses and in the course of typical plant development and growth [37].

As a result, we have determined that the HTT model is a practical way to represent the way in which environmental factors (Ψ and T) impact the germination of seeds in seed lots. The hydro-time constant (HT) determined for lentil was 96.77 (MPa Ch^− 1^) as shown in Table 3. In comparison to high T and low T, seed germination agronomic parameters including G%, TGI, GRI, GE, GI, SVI-II, and SVI-I were diminished. It is the consequence of chemical and cellular processes within the embryo that are thermo-inhibited. Based on the statistical analysis, the cardinal temperatures and θHTT provide a comprehensive explanation for the interaction effect of T and Ψ on the seed germination population.

The effect of temperature on other legumes is similar to that of lentils [38]. also reported arrow leaf clover germination was negatively affected at high temperatures, achieving 17 and 9% germination at day/night temperature treatments of 30/20 and 35/25°C, respectively. Button medic, Tifton burr medic, alfalfa, and crimson clover had the greatest germination of all entries at low (5 °C) temperatures. Germination at 35 °C was minimal, except for alfalfa and hairy vetch. 600RR alfalfa had the highest germination rate at 75%. Little burr medic, burr medic, and arrowleaf clover were particularly sensitive to high temperatures (30 °C), resulting in the lowest germination rates among all cool-season legume entries.

According to [39], crimson clover germinates well and quickly in all day/night temperature treatments ranging from 15/5 to 35/25°C. However, after 12 days at 4.5°C, ‘Yuchi’ arrowleaf and ‘Talladega’ red clovers had germination rates of more than 80% and about 20%, respectively. These studies found that high temperatures had a negative influence on bean germination, which is consistent with our findings. In the study, it is reported [40] that the final germination percentage of several annual Trifolium spp. remained constant between 5 and 20 °C, but decreased to zero as temperatures increased. On the other hand, the final germination percentage of perennial Trifolium spp. was constant from 5 to 30 °C and only declined at 35 °C. Another study measuring germination of several accessions of Medicago and Trifolium spp. found no difference in total germination between 5 and 20 °C, but there was considerably reduced germination at 0.5 and 30 °C. According to [41], the optimal temperature range for the germination of 15 accessions from six Vicia species is 18 to 23 °C. Other investigations have found variances in species germination percentage responses to temperature.

## Conclusions

The changing temperatures and water potentials had a significant impact on the germination characteristics. The highest hydro-time constant (θH) of 105.12 was recorded at 25 °C, while the lowest of 23.52 was observed at 40 °C. Additionally, the base, optimum, and ceiling temperatures were determined as 15 °C, 30 °C, and 40 °C, respectively. The preservation of enzymatic activity serves as a crucial protective mechanism against damage caused by oxidative stress. The characteristics of germination may deteriorate as energy is allocated towards anti-stress mechanisms (antioxidant enzymes) that are indispensable for neutralizing reactive oxygen species (ROS) produced during mitochondrial respiration at the germination stage. Such studies can facilitate the establishment of the optimal temperature and water potential for the germination of crop species, as well as the comprehension of the adaptive response mechanisms during the early developmental stage of a plant, which is the most vulnerable phase. Nevertheless, the intricate physiological, biochemical, and molecular responses of the tested seed populations to abiotic factors should be meticulously considered in the model’s parameters for predicting future germination times.

## Data Availability

All data generated or analysed during this study are included in this published article.
